# Impaired Myostatin Function Alters Body Composition Without Detectable Attenuation of Imiquimod-Induced Psoriasiform Dermatitis

**DOI:** 10.3390/ijms27198518

**Published:** 2026-09-24

**Authors:** Yoshihiro Matsuda, Shin Morizane, Yuma Sakamoto, Nobuyasu Baba, Masanori Iseki, Daiki Takezaki, Yoshio Kawakami, Shin-Ichiro Nishimatsu, Yutaka Ohsawa, Yoshihide Sunada, Tomoyuki Mukai

**Affiliations:** 1 Department of Dermatology, Graduate School of Medicine, Dentistry and Pharmaceutical Sciences, Okayama University, Okayama 700-8558, Japan; p125773v@s.okayama-u.ac.jp (Y.M.);; 2 Department of Immunology and Molecular Genetics, Kawasaki Medical School, Kurashiki 701-0192, Japan; 3 Laboratory of Immunology, Faculty of Veterinary Medicine, Okayama University of Science, Imabari 794-8555, Japan; 4 Department of Natural Sciences, Kawasaki Medical School, Kurashiki 701-0192, Japan; 5 Department of Neurology, Kawasaki Medical School, Kurashiki 701-0192, Japan

**Keywords:** psoriasis, myostatin, muscle hypertrophy, obesity, imiquimod, high-fat diet

## Abstract

Psoriasis is a chronic inflammatory skin disease closely associated with obesity and metabolic dysfunction. While excess adiposity is considered a key risk factor, the independent contribution of body composition to psoriatic inflammation remains unclear. Myostatin negatively regulates skeletal muscle growth, and its inhibition leads to muscle hypertrophy and reduced adiposity. In this study, we investigated whether altered body composition due to impaired myostatin function affects imiquimod (IMQ)-induced psoriasiform dermatitis. Wild-type (WT) and dominant-negative myostatin transgenic mice were fed either a normal diet or a high-fat diet (HFD) for 10 weeks, followed by topical IMQ treatment to induce dermatitis. Mutant mice exhibited increased skeletal muscle mass and reduced visceral fat compared with WT controls, indicating marked changes in body composition. In WT mice, HFD feeding increased fat mass and tended to aggravate IMQ-induced ear swelling. In contrast, no significant effect of impaired myostatin function on ear thickness, epidermal hyperplasia, or the expression of psoriasis-associated genes was detected, despite reduced visceral fat. Under the present experimental conditions, these changes in body composition were not associated with a detectable attenuation of psoriasiform skin inflammation. Our results suggest that obesity-related psoriasis is influenced by metabolic and immunological factors beyond body composition.

## 1. Introduction

Psoriasis is a chronic immune-mediated inflammatory skin disease affecting approximately 2–3% of the population and is frequently associated with systemic comorbidities, particularly obesity and metabolic syndrome [1,2]. Obesity has been identified as an independent risk factor for psoriasis incidence and severity, and weight reduction has been associated with improvement in disease activity in clinical studies [3,4,5]. These findings suggest a close link between metabolic status and psoriatic inflammation.

Adipose tissue acts as an active endocrine organ that produces pro-inflammatory cytokines and adipokines capable of enhancing Th17-mediated immune responses, a central pathway in psoriasis pathogenesis [6]. In addition to increased fat mass, obesity-associated metabolic abnormalities such as dyslipidemia and insulin resistance may further contribute to systemic inflammation [7,8]. Nevertheless, the specific contribution of excess adiposity itself to psoriatic inflammation remains unclear.

Skeletal muscle is another major metabolic organ that interacts with adipose tissue and systemic inflammatory processes [9]. Physical exercise has been reported to improve psoriasis severity in both clinical and experimental settings [10,11,12]. However, it remains unclear whether the beneficial effects of exercise are attributable to exercise itself or to secondary alterations in body composition, such as increased muscle mass and reduced fat mass.

Myostatin is a key negative regulator of skeletal muscle growth [13]. Genetic inhibition of myostatin signaling induces marked skeletal muscle hypertrophy and reduces adiposity, particularly visceral fat, without requiring physical exercise [14,15,16]. Myostatin loss-of-function mice provide a useful model for examining the effects of altered body composition on inflammation.

In the present study, we used dominant-negative myostatin transgenic mice as a genetic model characterized by increased skeletal muscle mass and reduced visceral adiposity. We examined whether these systemic alterations in body composition were associated with changes in the macroscopic, histological, and molecular features of imiquimod (IMQ)-induced psoriasiform dermatitis under normal diet (ND) and high-fat diet (HFD) conditions.

## 2. Results

### 2.1. Generation of a Psoriasiform Dermatitis Model in Mutant Myostatin Transgenic Mice Under HFD Conditions

To investigate the effects of altered body composition on psoriasiform dermatitis, we combined an HFD-induced obesity model with a genetic model of muscle hypertrophy caused by impaired myostatin function (Figure 1). Wild-type (WT) mice and dominant-negative myostatin transgenic (Mut) mice were fed either ND or HFD for 10 weeks starting at 6 weeks of age. At 15 weeks of age, psoriasiform dermatitis was induced by daily topical application of IMQ to the ear for seven consecutive days. Petrolatum-treated mice served as controls.

This design generated eight experimental groups defined by genotype (WT vs. Mut), diet (ND vs. HFD), and topical treatment (petrolatum vs. IMQ). One day after the final treatment, mice were euthanized and tissues were collected for analysis.

### 2.2. Effects of HFD and Impaired Myostatin Function on Body Composition

We first examined whether impaired myostatin function altered body composition under ND and HFD conditions. Body weight and body weight gain during the feeding period showed modest increases in HFD-fed mice compared with ND-fed mice (Figure 2a–c). Consistent with the role of myostatin in regulating skeletal muscle growth, Mut mice exhibited increased skeletal muscle mass compared with WT mice, as evidenced by the greater muscle bulk in the posterior hindlimb of Mut mice (Figure 2d). Notably, the weight of the gastrocnemius muscle, a fast-twitch muscle, was significantly increased in Mut mice, whereas the weight of the soleus muscle, which is enriched in slow-twitch fibers, was not significantly altered (Figure 2e,f). This muscle-type–specific effect is consistent with previously reported phenotypes of myostatin loss-of-function models [17].

Epididymal fat mass was significantly increased by HFD feeding but remained smaller in Mut mice than in WT mice under both ND and HFD conditions (Figure 2g,h). These findings indicate that impaired myostatin function promotes skeletal muscle hypertrophy and attenuates visceral fat accumulation under HFD conditions. Although significant sex-related interactions were detected for gastrocnemius muscle weight in the IMQ-treated groups, sex-stratified analyses confirmed the overall genotype-associated increase in muscle mass in both sexes (Figure S1; Table S2).

### 2.3. Effects of HFD and Impaired Myostatin Function on Systemic Metabolic Parameters

We next evaluated systemic metabolic parameters. HFD feeding increased serum total cholesterol levels compared with ND groups (Figure 3a). Triglyceride and glucose levels also tended to be higher in HFD-fed mice, although the magnitude of these changes varied across groups (Figure 3b,c). Serum adiponectin levels showed relatively small differences among groups (Figure 3d). We then examined whether impaired myostatin function influenced these metabolic parameters. Comparison between WT and Mut mice revealed no substantial differences in serum lipid levels, glucose levels, or adiponectin concentrations under either ND or HFD conditions. A sex-related interaction was detected for serum triglyceride levels in the petrolatum-treated groups, although this did not alter the overall interpretation of the systemic metabolic findings; detailed analyses are provided in the Supplementary Materials (Table S2 and Figure S2).

Overall, these findings indicate that HFD feeding induced metabolic alterations consistent with an obesity-associated phenotype, whereas no consistent differences in systemic metabolic parameters associated with impaired myostatin function were detected.

### 2.4. Effects of HFD and Impaired Myostatin Function on IMQ-Induced Psoriasiform Dermatitis

We next evaluated whether HFD and impaired myostatin function influenced the severity of IMQ-induced psoriasiform dermatitis. Ear thickness was measured daily during the IMQ treatment period.

In petrolatum-treated groups, only minor changes in ear thickness were observed (Figure 4a). In IMQ-treated groups, IMQ application induced progressive ear swelling consistent with psoriasiform dermatitis (Figure 4b). Mixed-effects analysis revealed significant effects of time (*p* < 0.0001) and group (*p* = 0.0052), as well as a significant time × group interaction (*p* < 0.0001). Subsequent multiple-comparison analysis showed that ear thickness was significantly greater in the WT-HFD group than in the WT-ND group on days 4–7 (Figure 4b). Comparison between WT and Mut mice revealed no significant difference in the magnitude of ear thickening under either ND or HFD conditions (Figure 4b). Exploratory sex-stratified mixed-effects analyses showed significant group and time × group effects in females but not in males; however, these analyses did not constitute a formal test of sex interaction (Figure S3).

These results suggest that HFD tends to aggravate IMQ-induced skin inflammation, particularly during the evolving phase of dermatitis, whereas no significant effect of impaired myostatin function on overall dermatitis severity was detected under the present experimental conditions.

### 2.5. Histological Analysis of Psoriasiform Skin Lesions

Histological analysis of ear tissue was performed to assess epidermal hyperplasia and overall tissue morphology.

IMQ-treated mice exhibited epidermal hyperplasia accompanied by dermal inflammatory changes (Figure 5a). Pairwise comparisons with the corresponding petrolatum-treated controls confirmed that IMQ treatment significantly increased epidermal thickness in all genotype/diet combinations (Figure 5b). Consistent with the ear thickness measurements, HFD feeding tended to increase epidermal thickness, whereas no significant effect of impaired myostatin function on epidermal thickness was detected under the present experimental conditions (Figure 5b). Similar findings were obtained in exploratory sex-stratified analyses of epidermal thickness (Figure S4).

### 2.6. Expression of Psoriasis-Associated Genes in Ear Tissue

To determine whether HFD and impaired myostatin function influence local inflammatory responses, we analyzed the expression of genes associated with psoriasis.

In petrolatum-treated groups, the expression levels of inflammatory genes were generally low (Figure 6). IMQ treatment markedly increased the expression of several cytokines and chemokines associated with psoriatic inflammation, including *Tnfa*, *Il23a*, *Il17a*, *Il22*, and *Ccl20*, although the statistical significance varied among genes and genotype/diet combinations (Figure 6). HFD feeding produced modest and variable changes in selected inflammatory transcripts; however, these changes were not accompanied by a consistent alteration across the psoriasis-associated gene panel.

We also evaluated keratin 16 (*Krt16*), a marker of keratinocyte activation and epidermal hyperproliferation in psoriatic skin. IMQ treatment markedly upregulated *Krt16* expression, whereas neither HFD feeding nor impaired myostatin function further enhanced its expression.

Overall, no consistent effect of impaired myostatin function on the expression of psoriasis-associated inflammatory cytokines or keratinocyte activation markers was detected in IMQ-treated skin.

To further characterize local inflammatory responses, we additionally examined *Il10* expression. *Il10* expression tended to be higher in IMQ-treated groups than in petrolatum-treated groups, but no consistent additional alteration associated with impaired myostatin function or HFD feeding was observed (Figure S5). We also examined genes associated with TLR7 downstream signaling, including *Irf7* and *Isg15*. Neither gene showed a consistent alteration associated with impaired myostatin function or HFD feeding (Figure S5). The oxidative stress-responsive genes *Hmox1* and *Nqo1* were also assessed, and neither showed a consistent alteration associated with impaired myostatin function or HFD feeding (Figure S5).

## 3. Discussion

In the present study, impaired myostatin function, which induced skeletal muscle hypertrophy and reduced visceral adiposity, was not associated with a significant attenuation of IMQ-induced psoriasiform dermatitis under either ND or HFD conditions. Thus, despite marked changes in body composition, we did not detect a corresponding improvement in psoriasis-like skin inflammation under the present experimental conditions. These findings suggest that factors beyond changes in body composition may contribute to obesity-associated psoriasiform inflammation.

Obesity is a well-established risk factor for psoriasis and is associated with chronic low-grade inflammation, altered lipid metabolism, and dysregulated adipokine secretion [3,4,6]. In the present study, HFD feeding tended to exacerbate psoriasiform dermatitis, consistent with previous reports [12,18,19]. In the longitudinal analysis, the difference in ear swelling between WT-HFD and WT-ND mice was detected on days 4–7, indicating that the effect of HFD was time-dependent rather than uniform throughout the experimental period. However, the reduction in visceral fat mass associated with impaired myostatin function was not accompanied by a detectable improvement in disease severity, suggesting that changes in visceral adiposity alone may not fully account for psoriasiform inflammation in this model. These findings provide a new perspective on the link between obesity and psoriasis. Although multiple mechanisms, including fat accumulation, adipokine dysregulation, and metabolic abnormalities, have been proposed to explain obesity-associated worsening of psoriasis, our results suggest that changes in adiposity alone do not appear to fully account for disease severity.

Dietary fat itself may contribute to disease exacerbation. HFD alters circulating fatty acids and lipid signaling pathways that influence immune responses and keratinocyte activation [20]. Saturated fatty acids such as palmitic acid enhance inflammatory signaling and synergize with cytokines including TNF-α and IL-17A [21]. Notably, HFD-induced aggravation of psoriasiform dermatitis has been reported even without severe obesity, whereas genetically obese mice (*ob/ob* mice) fed a normal diet do not necessarily show similar exacerbation [19]. Free fatty acids can also promote early inflammatory responses independently of obesity [22]. Clinical studies indicate that fatty acid composition, rather than total levels, is altered in psoriasis patients [23,24]. Consistent with this concept, serum lipid parameters were comparable between WT and mutant myostatin transgenic mice, suggesting that reduced fat mass alone may not capture the metabolic changes relevant to inflammation. However, because targeted lipidomic and free fatty acid analyses were not performed in the present study, the contribution of specific lipid species or fatty acid profiles cannot be determined from our data.

Beyond dietary lipid effects, our previous study has shown that obesity and dyslipidemia synergistically aggravate psoriatic inflammation via metabolic mediators such as leptin and palmitic acid [21]. Metabolic comorbidities such as metabolic dysfunction-associated steatohepatitis (MASH) can also worsen psoriasis independently of obesity [18]. In the present study, serum adiponectin levels were similar between genotypes despite reduced adiposity in mutant mice, raising the possibility that factors related to adipose tissue quality, in addition to adipose tissue quantity, may be relevant to disease severity.

Our findings contrast with our previous report that aerobic treadmill exercise attenuated epidermal hyperplasia in obesity-associated psoriasiform dermatitis [12]. In that study, treadmill exercise improved skin pathology despite minimal effects on body weight or adiposity in an IMQ-induced psoriasiform dermatitis model. This observation is consistent with the possibility that the protective effects of exercise involve mechanisms beyond fat reduction. Exercise is known to induce systemic adaptations, including improved metabolic function, anti-inflammatory effects, and the release of muscle-derived factors (myokines or exerkines) [25,26,27]. In contrast, impaired myostatin function increased muscle mass without reproducing the beneficial effects of physical activity on psoriasiform skin changes. Together, these observations raise the possibility that muscle quantity and muscle activity have distinct relationships with systemic inflammation. Although impaired myostatin function increased muscle mass, it may not reproduce the exercise-induced secretory and metabolic remodeling that could contribute to the attenuation of psoriasiform skin changes. However, the present study used a 7-day IMQ protocol, whereas our previous exercise study used a 5-day protocol. Differences in IMQ exposure duration may affect the kinetics and magnitude of skin inflammation, and the longer IMQ exposure in the present study may have reduced the ability to detect subtle systemic effects. Therefore, the findings of the two studies should be compared qualitatively rather than directly on a quantitative basis.

Myostatin loss-of-function mice preferentially develop fast-twitch glycolytic muscle hypertrophy, whereas endurance exercise promotes oxidative slow-twitch fibers [17,28]. These fiber types differ in metabolic capacity and inflammatory signaling. In the present study, hypertrophy was prominent in the gastrocnemius but not in the soleus, a pattern compatible with the preferential effects of myostatin loss of function on fast-twitch muscles reported previously [17]. However, muscle fiber composition was not directly assessed in the present study. Thus, differences in muscle fiber composition remain one possible explanation for the divergent findings between the present genetic model and our previous exercise intervention study.

Clinical studies have reported that sarcopenia is more prevalent in patients with psoriasis than in the general population, suggesting a possible link between muscle loss and disease severity [29]. This association has been interpreted as evidence that reduced muscle mass may contribute to systemic inflammation. However, in the present model, increased muscle mass was not associated with a detectable improvement in psoriasiform inflammation, suggesting that muscle function, metabolic activity, or exercise-related adaptations may be more relevant than muscle quantity alone.

Limitations of this study should be considered when interpreting these findings. First, the IMQ-induced psoriasiform dermatitis model represents acute inflammation and may not fully recapitulate the chronic and relapsing nature of human psoriasis. The strong innate immune activation induced by IMQ could potentially mask moderate systemic effects resulting from alterations in body composition. Future studies using milder or more chronic inflammatory models may help determine whether reductions in visceral adiposity exert protective effects under conditions in which the inflammatory stimulus is less dominant. Second, although formal interaction analyses were performed to evaluate potential sex-dependent effects, the sex-specific subgroup sizes were relatively small and unequal, particularly in the Mut/HFD/petrolatum female group (*n* = 2). Therefore, the study had limited statistical power to characterize sex-dependent effects, and the sex-stratified findings should be regarded as exploratory. Accordingly, male and female data were pooled for the primary analyses when no significant sex-related interaction was detected, whereas the sex-stratified analyses were considered exploratory. Third, immune cell infiltration in the affected skin, including populations such as neutrophils and γδ T cells, was not directly characterized by flow cytometry or immunohistochemistry. Therefore, we cannot exclude the possibility that impaired myostatin function or HFD altered specific infiltrating immune cell populations without producing detectable changes in the macroscopic, histological, or whole-tissue gene-expression analyses performed in this study. Fourth, muscle fiber composition, fatty acid composition, and muscle-derived factors, including candidate myokines, were not directly evaluated. Future studies measuring circulating and/or tissue levels of candidate myokines will be required to determine whether exercise-related humoral factors contribute to the observed differences between the two models.

Taken together, our findings show that the increase in skeletal muscle mass and reduction in visceral fat associated with impaired myostatin function were not accompanied by a detectable attenuation of psoriasiform inflammation under the present experimental conditions. These results suggest that obesity-associated psoriasis may involve complex metabolic–immunological interactions that extend beyond adiposity alone.

## 4. Materials and Methods

### 4.1. Mutant Myostatin Transgenic Mice

Mutant myostatin transgenic mice were generated as previously described [17,30]. These mice overexpress a mutant form of myostatin corresponding to the deletion mutation identified in Belgian Blue cattle. This mutation consists of an 11-nucleotide deletion that causes a frameshift and produces a premature stop codon, resulting in a truncated myostatin protein with impaired biological activity. The mutant myostatin acts in a dominant-negative manner and leads to increased skeletal muscle mass, consistent with the role of myostatin as a negative regulator of skeletal muscle growth [14].

For the present study, mutant myostatin transgenic mice originally generated on a mixed genetic background were backcrossed with C57BL/6J mice for more than 10 generations to obtain a congenic C57BL/6J genetic background. Hereafter, these mice are referred to as Mut.

### 4.2. Animals and Diets

Wild-type and Mut littermate mice were maintained under controlled environmental conditions (22 °C, 12 h light/dark cycle) at the animal facility of Kawasaki Medical School (Kurashiki, Japan). Animals were housed in groups of three to five per cage and had free access to food and water throughout the study.

Mice were randomly allocated to either a normal diet or a high-fat diet. The ND groups received standard laboratory chow (MF diet; Oriental Yeast Co., Tokyo, Japan), whereas the HFD groups were fed HFD32 (CLEA Japan, Tokyo, Japan). Dietary intervention was carried out for 10 weeks, from 6 to 16 weeks of age.

### 4.3. IMQ-Induced Psoriasiform Dermatitis Model

A psoriasiform dermatitis model was generated by topical treatment with 5% IMQ cream (Beselna; Mochida Pharmaceutical, Tokyo, Japan), following previously reported protocols [31]. Briefly, 25 mg of the cream was applied to the right ear once daily for seven consecutive days beginning at 15 weeks of age. Control animals received petrolatum using the same application procedure. Ear thickness was measured daily using a digital thickness gauge (PK-1012APX; Mitutoyo, Kawasaki, Japan). A 7-day treatment period was selected to assess the effects of impaired myostatin function over a sustained course of IMQ-induced inflammation.

### 4.4. Study Design

Mice were divided into eight experimental groups based on genotype (WT or Mut), diet (ND or HFD), and topical treatment (petrolatum or IMQ). A schematic overview of the experimental timeline is shown in Figure 1. From 6 weeks of age, mice assigned to HFD groups were fed a HFD. At 15 weeks of age, mice were subjected to daily topical treatment with either petrolatum or IMQ for a total of seven days (*n* = 7–12 per group). Both male and female mice were included in each group. The sex distribution was as follows (male/female): WT/ND/petrolatum (6/5), Mut/ND/petrolatum (4/5), WT/HFD/petrolatum (5/6), Mut/HFD/petrolatum (5/2), WT/ND/IMQ (6/6), Mut/ND/IMQ (5/3), WT/HFD/IMQ (6/6), and Mut/HFD/IMQ (5/3). Mice were euthanized one day after the final IMQ treatment, and blood and tissue samples were collected. The gastrocnemius, soleus, and epididymal fat tissues were weighed. Body weight was measured at 6 and 16 weeks of age, and body weight gain over the 10-week feeding period was calculated. Potential sex-dependent effects were additionally evaluated using formal interaction analyses as described in Section 4.9. For outcomes without significant interactions involving sex, male and female data were pooled for the primary presentation. Outcomes showing significant sex-related interactions were further examined using sex-stratified analyses presented in the Supplementary Materials.

### 4.5. Biochemical Assays

Serum levels of total cholesterol and triglycerides were quantified using a dry chemistry analyzer (SPOTCHEM EZ SP-4430; Arkray, Kyoto, Japan) in accordance with the manufacturer’s protocol. Blood glucose levels were measured with a LAB Gluco device (Foracare Japan Co., Tokyo, Japan).

### 4.6. ELISA for Serum Adipokines

Serum adiponectin concentrations were assessed using a DuoSet ELISA kit (R&D Systems, Minneapolis, MN, USA) according to the manufacturer’s protocol.

### 4.7. Histological Analysis of Ear Tissue

Histological examination of ear tissue was performed following previously reported methods [12]. Ear specimens were fixed in 4% paraformaldehyde prepared in phosphate-buffered saline for 24 h, then embedded in paraffin, sectioned, and stained with hematoxylin and eosin. Histological images were obtained using a VS100 slide scanner (Olympus, Tokyo, Japan) and subsequently evaluated with OlyVIA software (version 4.1; Olympus). Epidermal thickness, excluding the stratum corneum, was quantified in ten randomly selected regions per mouse using ImageJ software (ver. 1.54d; National Institutes of Health, Bethesda, MD, USA).

### 4.8. Real-Time Quantitative PCR

Real-time quantitative PCR (qPCR) analysis was performed according to previously reported methods [32,33]. Total RNA was isolated from ear tissue using RNAiso Plus (Takara Bio, Kusatsu, Japan) and reverse-transcribed into cDNA using the PrimeScript RT Reagent Kit (Takara Bio). Quantitative PCR was conducted with TB Green PCR Master Mix (Takara Bio) on a QuantStudio 1 System (Thermo Fisher Scientific, Waltham, MA, USA). Gene expression levels were calculated using the ΔΔCt method and normalized to *Gapdh*. Comparable results were obtained when *Hprt* was used as an alternative reference gene. Primer sequences are provided in Table S1. All reactions showed single-peak dissociation curves, confirming the specificity of amplification.

### 4.9. Statistical Analysis

Data are shown as mean values with individual data points. For ear thickness measurements, time-course data are presented as mean ± standard error of the mean (SEM). Longitudinal ear-thickness data were analyzed using a mixed-effects model with restricted maximum likelihood (REML), with time treated as a repeated-measures factor and individual mouse as a random effect. The model included the effects of time and group and the time × group interaction. Post hoc pairwise comparisons among groups at individual time points were performed using Tukey’s multiple-comparison test. For longitudinal ear-thickness measurements, exploratory sex-stratified mixed-effects analyses were also performed using the same model.

For single-time-point outcomes, comparisons among the four genotype/diet groups were performed separately within the petrolatum-treated and IMQ-treated groups using the Kruskal–Wallis test followed by Dunn’s multiple-comparison test, as appropriate. To directly evaluate the effect of IMQ treatment, additional pairwise comparisons between petrolatum- and IMQ-treated mice were performed within each genotype/diet combination (WT-ND, WT-HFD, Mut-ND, and Mut-HFD) using the Mann–Whitney U test. *p* values from these four petrolatum-versus-IMQ comparisons for each outcome were adjusted for multiple testing using the Holm–Šídák method. Because the primary analytical questions concerned genotype- and diet-related differences within each topical-treatment condition and the effect of IMQ within each genotype/diet combination, these comparisons were analyzed separately rather than in a single global genotype × diet × treatment factorial model. Thus, genotype × diet × treatment interactions were not formally estimated in the primary analysis.

To evaluate potential sex-dependent modification of genotype and diet effects, three-way ANOVA with sex, diet, and genotype as factors was additionally performed separately within the petrolatum-treated and IMQ-treated groups for single-time-point outcomes. Sex × diet, sex × genotype, and sex × diet × genotype interactions were evaluated. When a significant interaction involving sex was detected, additional sex-stratified analyses were performed using two-way ANOVA with diet and genotype as factors, followed by Tukey’s multiple-comparison test for prespecified pairwise comparisons. These sex-stratified analyses were considered exploratory because of the relatively small and unequal subgroup sizes.

All analyses were conducted with GraphPad Prism version 10 (GraphPad Software, San Diego, CA, USA). A *p* value of less than 0.05 was considered statistically significant.

## Figures and Tables

**Figure 1:**
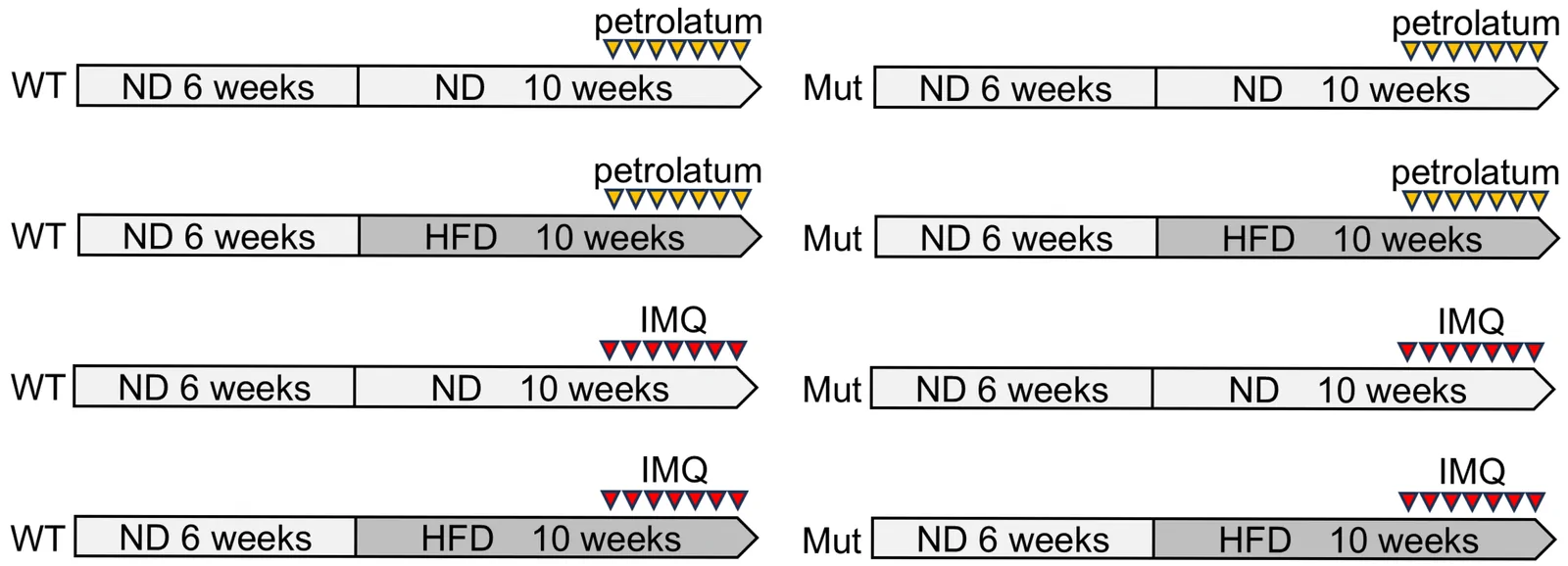
Generation of a murine experimental model combining impaired myostatin function, high-fat diet (HFD), and psoriasiform dermatitis. Study design: Six-week-old wild-type (WT) and mutant myostatin transgenic (Mut) mice were fed a normal diet (ND) or a HFD for 10 weeks from 6 to 16 weeks of age. Imiquimod (IMQ) cream (25 mg/mouse) was topically applied to the ear for seven consecutive days (*n* = 7–12 per group). Petrolatum was applied using the same protocol for comparison. One day after the last treatment, blood, skin, epididymal fat, and muscle samples were collected. Yellow and red triangles indicate daily applications of petrolatum and IMQ, respectively.

**Figure 2:**
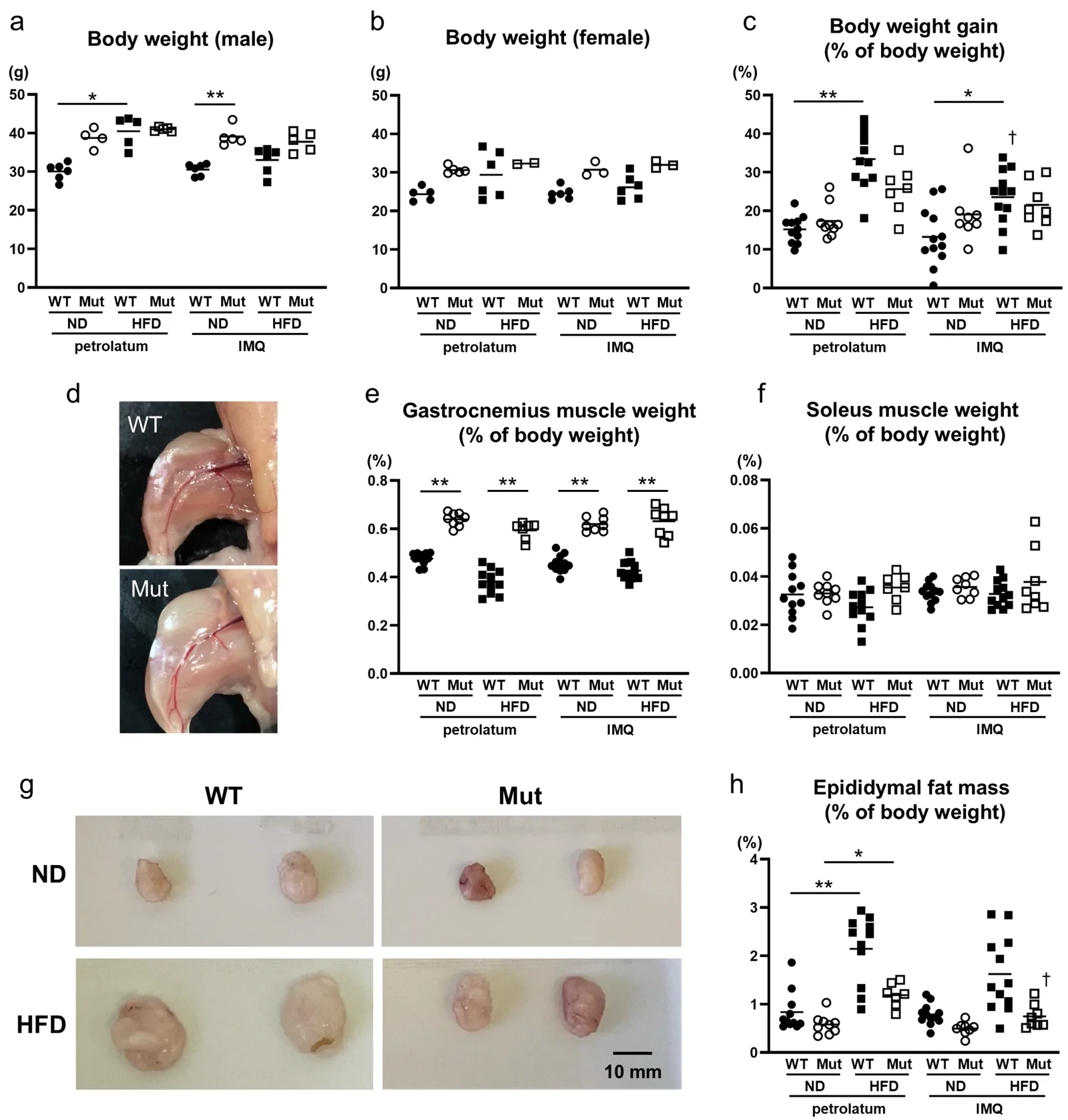
The effects of impaired myostatin function and a high-fat diet (HFD) on body composition. (**a**,**b**) Body weights of male (**a**) and female (**b**) mice. (**c**) Body weight gain of mice. Body weight gain was calculated as the percentage change relative to body weight at 6 weeks of age. (**d**) Gross morphology of the hindlimb (thigh region) in wild-type (WT) and mutant myostatin transgenic (Mut) mice. Representative macroscopic images obtained after skin reflection to expose subcutaneous tissue. (**e**,**f**) Weights of gastrocnemius muscle (**e**) and soleus muscle (**f**). (**g**) Representative macroscopic images of epididymal fat. (**h**) Weight of epididymal fat. Values are presented as individual dots with bars representing the mean. Black symbols indicate WT mice, and white symbols indicate Mut mice. Circles represent normal diet (ND) groups, and squares represent HFD groups. * *p* < 0.05 and ** *p* < 0.01 vs. the indicated group, by the Kruskal–Wallis test followed by Dunn’s multiple-comparison test. † *p* < 0.05 vs. the corresponding petrolatum-treated group, by the Mann–Whitney U test with Holm–Šídák correction. IMQ, imiquimod.

**Figure 3:**
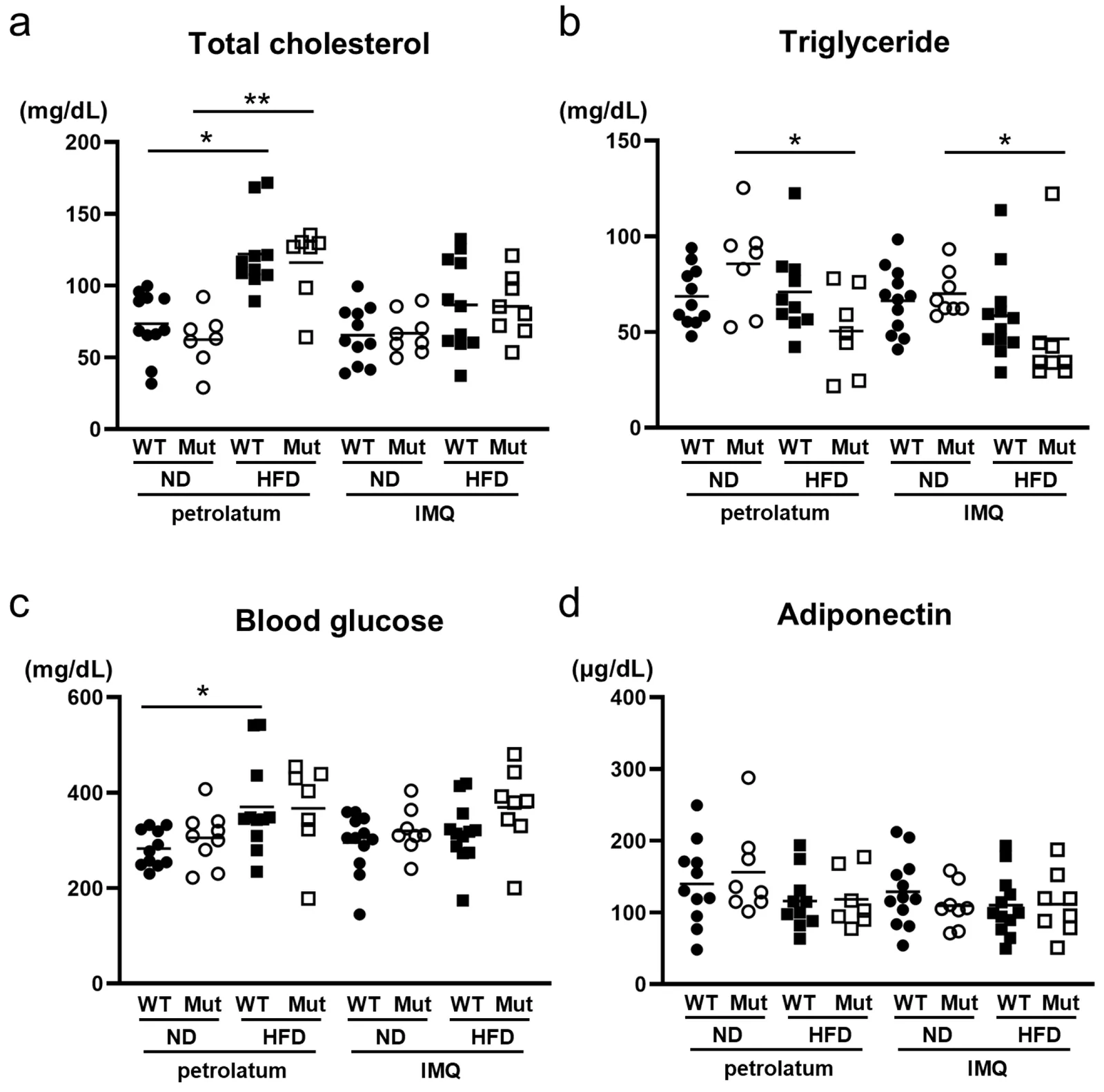
The effects of impaired myostatin function and a high-fat diet (HFD) on systemic metabolic parameters. Serum concentrations of total cholesterol (**a**) and triglycerides (**b**), as well as blood glucose levels (**c**), were determined using biochemical assays. Serum adiponectin concentrations (**d**) were determined by ELISA. Values are presented as individual dots with bars representing the mean. Black symbols indicate wild-type (WT) mice, and white symbols indicate mutant myostatin transgenic (Mut) mice. Circles represent normal diet (ND) groups, and squares represent HFD groups. * *p* < 0.05 and ** *p* < 0.01 vs. the indicated group, by the Kruskal–Wallis test followed by Dunn’s multiple-comparison test. IMQ, imiquimod.

**Figure 4:**
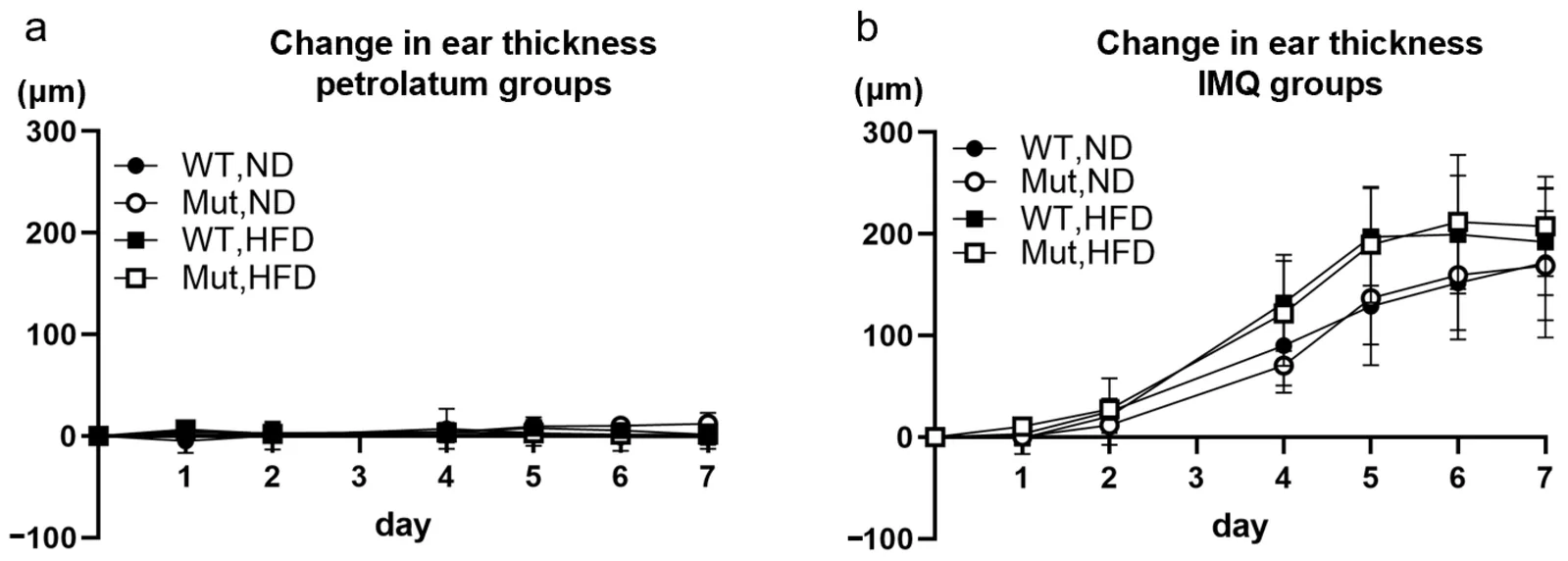
Changes in ear thickness in imiquimod (IMQ)-induced psoriasiform skin inflammation. (**a**,**b**) Changes in ear thickness. Wild-type (WT) and mutant myostatin transgenic (Mut) mice were fed a normal diet (ND) or a high-fat diet (HFD) and treated with IMQ cream (25 mg/mouse) or petrolatum (*n* = 7–12 per group). Ear thickness was measured daily using a digital thickness gauge. Lines show time-course changes in ear thickness. Values are presented as the mean ± standard error of the mean. Black symbols indicate WT mice, and white symbols indicate Mut mice. Circles represent ND groups, and squares represent HFD groups.

**Figure 5:**
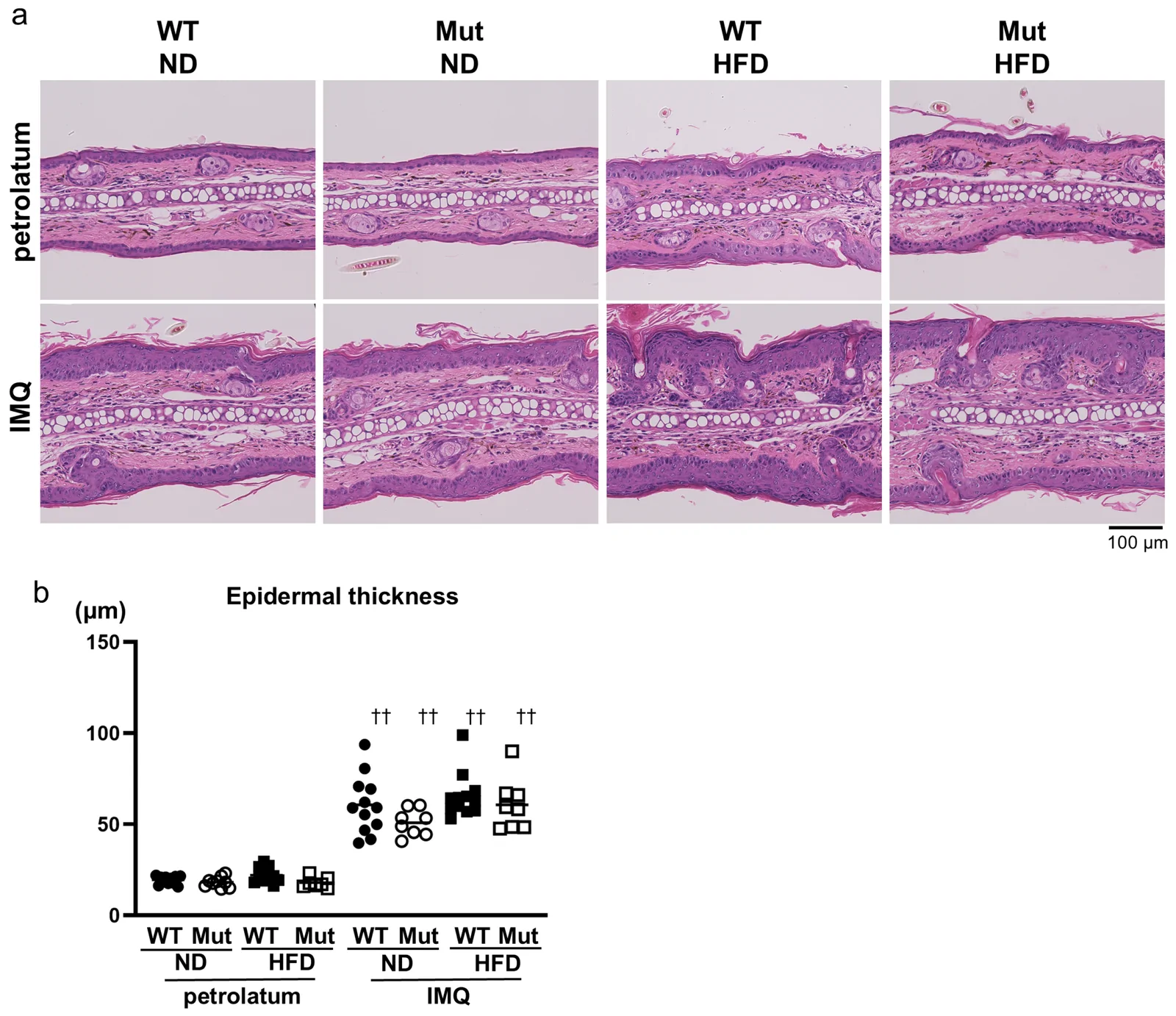
Histological analysis of psoriasiform skin changes. (**a**) Representative images of hematoxylin- and eosin-stained ear sections from the indicated mice. Bar = 100 μm. (**b**) Histological analysis of epidermal thickness. Values are presented as individual dots with bars representing the mean. Black symbols indicate wild-type (WT) mice, and white symbols indicate mutant myostatin transgenic (Mut) mice. Circles represent normal diet (ND) groups, and squares represent high-fat diet (HFD) groups. †† *p* < 0.01 vs. the corresponding petrolatum-treated group, by the Mann–Whitney U test with Holm–Šídák correction. IMQ, imiquimod.

**Figure 6:**
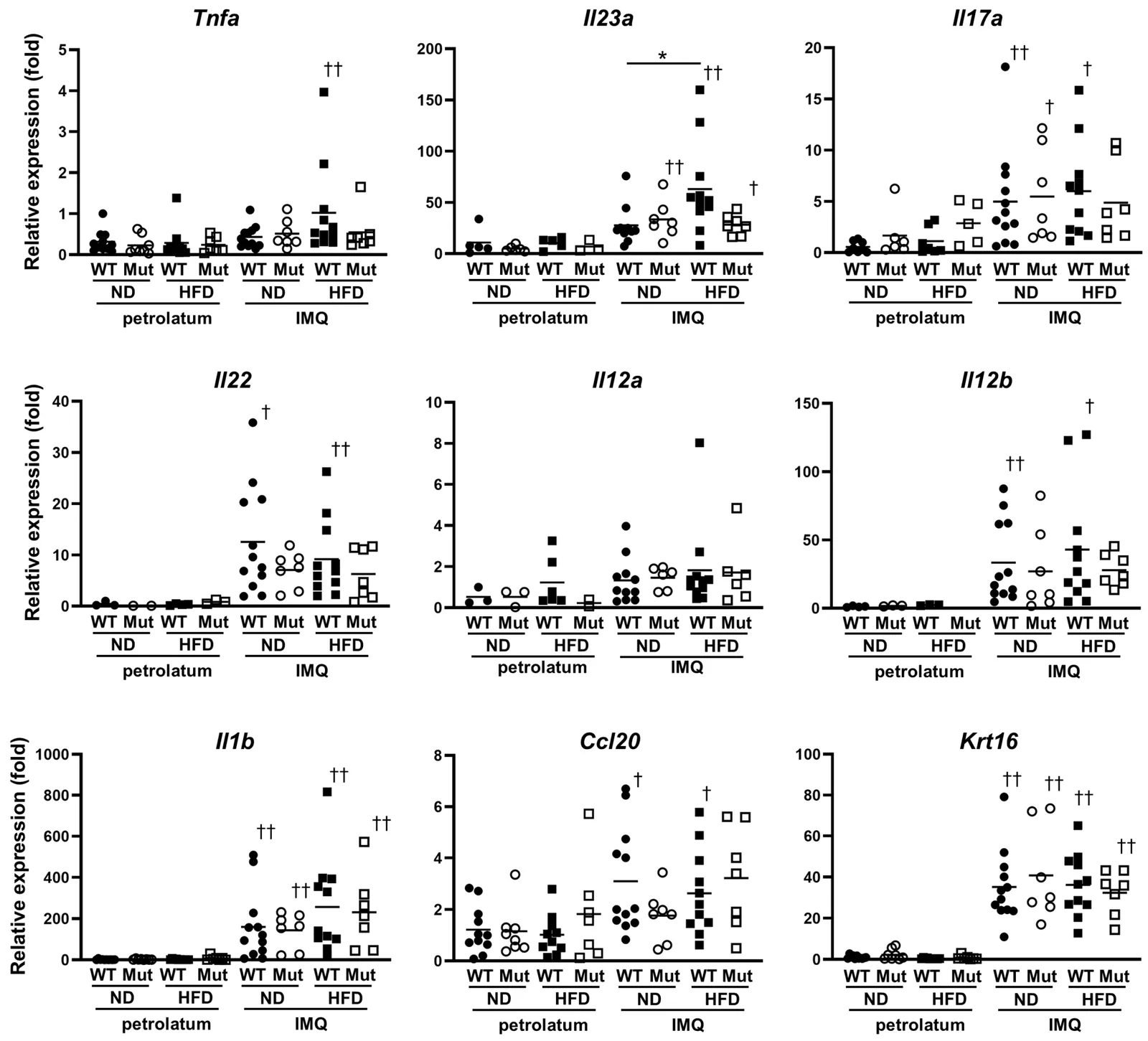
Alterations in the mRNA expression of psoriasis-associated genes in ear tissue. Total RNA was extracted from the ear tissues from the indicated mice. mRNA expression levels were determined by quantitative PCR. Expression levels were normalized to *Gapdh* and expressed relative to the ND-fed, petrolatum-treated WT group. Values are presented as individual dots with bars representing the mean. Samples in the petrolatum-treated groups with mRNA expression levels below the detection limit were excluded from the plots. Black symbols indicate WT mice, and white symbols indicate mutant myostatin transgenic (Mut) mice. Circles represent ND groups, and squares represent HFD groups. * *p* < 0.05 vs. the indicated group, by the Kruskal–Wallis test followed by Dunn’s multiple-comparison test. † *p* < 0.05, †† *p* < 0.01 vs. the corresponding petrolatum-treated group, by the Mann–Whitney U test with Holm–Šídák correction. IMQ, imiquimod.

## Data Availability

The data that support the findings of this study are available from the corresponding author upon reasonable request.

## Supplementary Materials

The following supporting information can be downloaded at: https://www.mdpi.com/article/10.3390/ijms27198518/s1.
