# Supplementary material for: Impaired Myostatin Function Alters Body Composition Without Detectable Attenuation of Imiquimod-Induced Psoriasiform Dermatitis
Source: Int J Mol Sci. 2026 Sep 24;27(19):8518. doi: 10.3390/ijms27198518 (PMC13665227; doi:10.3390/ijms27198518)
Supplement: Supplementary file 1 [file ijms-27-08518-s001.zip › Supple Table S1_20260912.pdf]

Table S1: qPCR primers used in this study

Gene	Gene accession number		Sequence	Amplicon size (bp)	Annealing temperature (°C)
Tnfa	NM_013693.2	Forward:<br>Reverse:	catcttctcaaaattcgagtgaca<br>tgggagtagacaaggtacaaccc	175	60
Il23a	NM_031252.2	Forward:<br>Reverse:	caccagcgggacatatgaa<br>ccttgtgggtcacaaccat	100	60
Il17a	NM_010552.3	Forward:<br>Reverse:	tgtgaaggtcaacctcaaagtc<br>gagggatatctatcagggtcttca	132	60
Il22	NM_016971.2	Forward:<br>Reverse:	tgacgaccagaacatccaga<br>aatcgccttgatctctccac	85	60
Il12a	NM_001159424.2	Forward:<br>Reverse:	ttccaacagcctcaccctc<br>ccaaaaagaggaggtagcgtgat	84	64
Il12b	NM_001303244.1	Forward:<br>Reverse:	aaggaacagtgggtgtccag<br>gttagcttctgaggacacatcttg	74	64
Il1b	NM_008361.3	Forward:<br>Reverse:	agttgacggaccccaaaag<br>agctggatgctctcatcagg	75	60
Ccl20	NM_016960.2	Forward:<br>Reverse:	aactgggtgaaaagggctgt<br>gtccaattccatcccaaaaa	86	60
Krt16	NM_001313958.1	Forward:<br>Reverse:	atgagctgaccctgtccaga<br>ctcaaggcaagcatctcctc	106	60
Il10	NM_010548.2	Forward:<br>Reverse:	cagagccacatgctcctaga<br>tgtccagctggtcctttgtt	79	62
Hmox1	NM_010442.2	Forward:<br>Reverse:	tgcaggtgatgctgacagag<br>tctgcaggggcagtatcttg	103	60
Nqo1	NM_008706.5	Forward:<br>Reverse:	ctctggccgattcagagtgg<br>ctcccagacggtttccagac	147	60
Irf7	NM_016850.3	Forward:<br>Reverse:	cttcagcactttcttccgaga<br>tgtagtgtggtgacccttgc	68	60
Isg15	NM_015783.3	Forward:<br>Reverse:	agtcgacccagtctctgactct<br>ccccagcatcttcaccttta	143	60
Gapdh	NM_008084.2	Forward:<br>Reverse:	atcaagaaggtggtgaagca<br>gacaacctggtcctcagtgt	75	60
Hprt	NM_013556.2	Forward:<br>Reverse:	tcctcctcagaccgctttt<br>cctggttcatcatcgctaatc	90	60
