# Supplementary material for: Impaired Myostatin Function Alters Body Composition Without Detectable Attenuation of Imiquimod-Induced Psoriasiform Dermatitis
Source: Int J Mol Sci. 2026 Sep 24;27(19):8518. doi: 10.3390/ijms27198518 (PMC13665227; doi:10.3390/ijms27198518)
Supplement: Supplementary file 1 [file ijms-27-08518-s001.zip › Supple Table S2 202609014_1220.pdf]

Supplementary Table S2. Three-way ANOVA evaluating sex-related interactions

| Outcome | Treatment | Sex | Sex×Diet | Sex×Genotype | Sex×Diet×Genotype |
|---|---|---|---|---|---|
| Body weight | petrolatum | **<0.0001** | 0.1864 | 0.9996 | 0.2787 |
| Body weight | IMQ | **<0.0001** | 0.5893 | 0.6867 | 0.2844 |
| Body weight gain | petrolatum | 0.4686 | 0.7857 | 0.9965 | 0.4759 |
| Body weight gain | IMQ | 0.5032 | 0.2138 | 0.3471 | 0.8018 |
| Gastrocnemius muscle weight | petrolatum | 0.0546 | 0.6591 | 0.3366 | 0.5906 |
| Gastrocnemius muscle weight | IMQ | **<0.0001** | 0.6476 | **0.012** | **0.0203** |
| Soleus muscle weight | petrolatum | 0.8406 | 0.6219 | 0.8719 | 0.9251 |
| Soleus muscle weight | IMQ | 0.4609 | 0.708 | 0.8415 | 0.9233 |
| Epididymal fat mass | petrolatum | 0.8976 | 0.1012 | 0.148 | 0.1056 |
| Epididymal fat mass | IMQ | 0.6507 | 0.4203 | 0.0916 | 0.1852 |
| Total cholesterol | petrolatum | 0.1662 | 0.3887 | 0.088 | 0.1676 |
| Total cholesterol | IMQ | **<0.0001** | 0.52 | 0.1111 | 0.7162 |
| Triglyceride | petrolatum | 0.9563 | **0.0011** | 0.7852 | 0.1014 |
| Triglyceride | IMQ | 0.0981 | 0.3441 | 0.2478 | 0.0982 |
| Blood glucose | petrolatum | **0.0148** | 0.7704 | 0.9954 | 0.3713 |
| Blood glucose | IMQ | 0.509 | 0.8739 | 0.7291 | 0.8418 |
| Adiponectin | petrolatum | 0.3864 | 0.3389 | 0.8436 | 0.8145 |
| Adiponectin | IMQ | 0.4776 | 0.9369 | 0.7513 | 0.7513 |
| Epidermal thickness | petrolatum | **0.0389** | 0.7969 | 0.898 | 0.6834 |
| Epidermal thickness | IMQ | 0.2563 | 0.4031 | 0.5027 | 0.6687 |
| *Tnfa* | petrolatum | **0.0047** | 0.6462 | 0.789 | 0.8529 |
| *Tnfa* | IMQ | 0.3692 | 0.7168 | 0.1519 | 0.1148 |
| *Il23a* | petrolatum | 0.9047 | 0.4912 | 0.6405 | 0.9084 |
| *Il23a* | IMQ | 0.0756 | 0.3486 | 0.2781 | 0.0756 |
| *Il17a* | petrolatum | **0.026** | 0.4202 | 0.1924 | 0.4988 |
| *Il17a* | IMQ | 0.893 | 0.7764 | 0.7855 | 0.7667 |
| *Il22* | petrolatum | Not analyzed (insufficient sample size) | | | |
| *Il22* | IMQ | 0.6949 | 0.4424 | 0.4424 | 0.8822 |

| Outcome | Treatment | Sex | Sex×Diet | Sex×Genotype | Sex×Diet×Genotype |
|---|---|---|---|---|---|
| *Il12a* | petrolatum | 0.3423 | 0.1986 | 0.3655 | 0.0678 |
| *Il12a* | IMQ | 0.6903 | 0.669 | 0.4065 | 0.4915 |
| *Il12b* | petrolatum | Not analyzed (insufficient sample size) | | | |
| *Il12b* | IMQ | 0.4532 | 0.6403 | 0.8808 | 0.8233 |
| *Il1b* | petrolatum | 0.3268 | 0.7442 | 0.2401 | 0.2334 |
| *Il1b* | IMQ | 0.1028 | 0.1028 | 0.1176 | 0.0712 |
| *Ccl20* | petrolatum | 0.5421 | 0.5556 | 0.8189 | 0.1034 |
| *Ccl20* | IMQ | 0.3749 | 0.7072 | 0.6138 | 0.8434 |
| *Krt16* | petrolatum | 0.1034 | 0.895 | 0.7668 | 0.9351 |
| *Krt16* | IMQ | 0.2113 | 0.516 | 0.598 | 0.7002 |
| *Il10* | petrolatum | 0.8794 | 0.3506 | 0.9629 | 0.7811 |
| *Il10* | IMQ | 0.2179 | 0.8293 | 0.4532 | 0.2161 |
| *Hmox1* | petrolatum | 0.6035 | 0.8149 | 0.5857 | 0.8726 |
| *Hmox1* | IMQ | **0.0144** | 0.5296 | 0.5167 | 0.5167 |
| *Nqo1* | petrolatum | 0.0628 | 0.3607 | 0.5513 | 0.1968 |
| *Nqo1* | IMQ | 0.7636 | 0.2945 | 0.8238 | 0.6753 |
| *Irf7* | petrolatum | 0.0578 | 0.1108 | 0.1712 | 0.1907 |
| *Irf7* | IMQ | 0.0792 | 0.7133 | 0.3295 | 0.2451 |
| *Isg15* | petrolatum | 0.7413 | 0.8951 | 0.4772 | 0.053 |
| *Isg15* | IMQ | 0.4223 | 0.4227 | 0.5249 | 0.9487 |

*Il22* and *Il12b* in the petrolatum-treated groups were not analyzed because the number of evaluable samples was insufficient for three-way ANOVA.
